# Perspective of an advocate: a case and framework for research advocacy in Africa

**DOI:** 10.1186/1750-9378-8-S1-S4

**Published:** 2013-07-15

**Authors:** Mary J Scroggins

**Affiliations:** 1 In My Sister’s Care, Washington, DC, 20019, USA

## Abstract

In making an experience-based case for research advocacy in Africa and suggesting a framework for building it, this paper covers factors such as basic tenets of patient advocacy, key components and urgent needs in building strong research advocacy, concepts and approaches from which guidance might be taken, and the feasibility of its development and growth throughout the continent. Research advocacy is defined as the meaningful engagement of patient advocates and their representatives in the research system.

As the clinical research system in Africa is developing and gaining strength, this is an opportune time for research advocacy to form and take root as an embedded component in the research structures on the continent. That is, the current state of development of the research system and the simultaneous interest in and rise of patient advocacy bode well for the likelihood of developing robust research advocacy, suggesting its feasibility. Even so, several developments are urgently needed to build, shore up, and sustain a framework receptive to maximizing the influence of an active network of patient advocates—many training in the subspecialty of research advocacy—and a research structure that supports and embeds advocate engagement.

## Introduction

In providing a case and framework for research advocacy in Africa, this paper draws on the experience of and infrastructure for research advocacy in the United States of America (America), where it is an increasingly present and influential component of the research landscape, and on lessons learned and observations made over a 16-year period of active advocacy. Conclusions are also drawn from engagement with an incredible array of patients, survivors, caregivers, and others involved in advocacy and in the research system. (See Additional file 1, “A tribute to patients and survivors.”)

The modes of research advocacy throughout Africa will differ from their American counterparts. They will be aligned with country and regional norms, cultures, religions, languages, and support. However, they can be informed by the significance and uniqueness of contributions of those who provide patient perspectives in American cancer and research communities. Over nearly three decades, at least since the founding in 1986 of the cancer survivor-led National Coalition for Cancer Survivorship [1], the impact of patient advocacy has grown significantly. The essential framework in Africa—the structure on which advocacy will stand—can be built on principles that contribute to the success and influence of patient and research advocacy in America.

Although patient advocacy is not a new concept in Africa, it is a largely nascent one, with groups like the Breast Cancer Association of Nigeria, Tanzania 50 Plus Campaign (prostate cancer focused), and People Living with Cancer (an umbrella organization focused on South Africans living with cancer) dotting the health landscape. Such groups and the growing continental research capacity are bolstered through the work of organizations like the African Organisation for Research and Training in Cancer (AORTIC). Formed in 1983, AORTIC’s key objectives are “to further the research relating to cancers prevalent in Africa, support the management of training programs in oncology for health care workers, deal with the challenges of creating cancer control and prevention programs and raise public awareness of cancer in Africa” [2]. Advocacy can also be strengthened by looking to the considerable American experience with and literature on patient advocacy, cultural competence in research, and community-based participatory research (CBPR).

The 2006 World Cancer Declaration recognized the following: “*By 2020, more than 16 million new cancer cases and 10 million cancer deaths are expected annually. Seventy percent of these deaths will likely occur in developing countries that are unprepared to address their growing cancer burden*” [3]. These projections highlight the urgency for improved cancer control, management, and care and for “collective action.” Research advocates and other advocates can prove instrumental in denying these projections a more solid foothold.

## Discussion

### Basic tenets of research advocacy: making the case for it

Patient advocacy includes many subspecialties, a fairly new but influential one being research advocacy. It is useful to have a shared definition of a patient advocate to discuss the value of patient and research advocacy. The America National Cancer Institute (NCI) defines a patient advocate as “A person who helps a patient work with others who have an effect on the patient's health, including doctors, insurance companies, employers, case managers, and lawyers.…Cancer advocacy groups try to raise public awareness about important cancer issues, such as the need for cancer support services, education, and research. Such groups work to bring about change that will help cancer patients and their families” [4]. Incorporated in the litany of services and engagement is research advocacy, the meaningful engagement of patient advocates and their representatives in the research system, which is integral to changes in health care research and service delivery.

The case for research advocacy in Africa is straightforward. There is an urgent need for all stakeholders to converge, there is an opportunity for mutual benefit and growth as the research system takes form and begins to mature, and there are examples of advocacy-at-work from which Africa can learn and model. As in the American model, research advocacy will strengthen the research process, change the way researchers see patients and consider patient needs, and keep patients at the center of research thinking and conduct. Research and other patient advocates will:

• Put faces on the disease. Many researchers will have encountered only the most ill patients at their very worst.

• Give voice to all patients and survivors. Patient voices are unique, experience-based, and indispensable, and patients will need to have the courage to become public about issues considered private and often stigmatic.

• Ask questions specific to the lived experience of cancer.

• Create a sense of urgency, by their very presence.

• Form mutually respectful and beneficial partnerships and relationships with researchers. This is a significant challenge, perhaps even a barrier, in a research system that within some cultures has strict lines of separation along lines of authority, responsibility, and class.

• Provide hope for patients, as patients need the example of survival and peer support.

All of these will result in the conduct of research more focused on issues important to patients, more patient-friendly, and more likely to accrue, increasing the prospect of ultimate benefit to patients.

### *A* framework, not *the* framework

Given the case for research advocacy and the unusual opportunity for it to develop in concert with the research system on the continent, a possible framework, as opposed to the only workable structure, is suggested. This structure aligns with basic components of research advocacy as it is practiced in America. Groups and individuals should feel free to borrow liberally from whatever will work in country-, culture-, and population-specific settings. It is not necessary to reinvent the wheel, but the wheel must be usable on African roads and byways. Embedded in the wheel of American patient and research advocacy are concepts and models such as community-based participatory research (CBPR) and “cultural competence” in research. These concepts largely address often significant concerns related to culture-, language-, and population-specific differences.

The proposed framework is based on a partnership between advocates and researchers, a natural alliance of individuals with like goals and differing but complementary experience and skill sets. This alliance must be nurtured in Africa just as it continues to be nurtured and to mature in America through training, ongoing engagement, and growing mutual respect. CBPR, which is defined as “a collaborative approach to research that equitably involves all partners in the research process and recognizes the unique strengths that each brings” [5], asserts the importance of full community participation in all elements of the research process. In fact, the research topic must be one of importance to the community being researched, and the research findings must be reported to the community. There are numerous examples of CBPR’s adaption and success in America from which African countries can draw. One example is the Deep South Network for Cancer Control, a Cancer Network Program funded by the NCI Center to Reduce Cancer Health Disparities and dedicated to building on “established community and institutional capacity in order to eliminate cancer health disparities by conducting community-based participatory education, training and research” [6].

Research advocacy should be seen as critical for the most patient-cantered, accruable research, as modelled in CBPR. It is not, however, easy or for the faint of heart or for the easily discouraged, a short-term project, or free of costs. For example, it requires significant funding, time, and effort. It also requires: (i) dedicated advocates; (ii) a supportive advocacy network and research system; (iii) an evolving state of mutual respect and acceptance between advocates and researchers; (iv) ongoing training and preparation for advocates and researchers; and (v) private and public sector investment and support. To promote the growth and influence of a viable and active patient advocate network, the advocacy framework must be country-specific and African-led, with support from countries and regions with more longstanding and robust networks.

### Becoming an advocate

To provide ongoing research advocacy coverage, a pipeline of willing and able volunteers must emerge. This pipeline of advocates can come from any walk of life, with many research advocates beginning their efforts in the subspecialties of support, community outreach, education, political, and fundraising. Whatever their route of entry, they are everyday citizens, including:

• Patients, survivors, family members, and caregivers.

• Curious, willing learners with or without a science or medical background.

• Clear communicators and listeners with the ability to read, write, and/or speak in the language of the populations represented or served.

• Members of health advocacy groups or other activist efforts.

• Scientists and clinicians personally affected by cancer.

• Other interested individuals with the time, lifestyle ability, and commitment to ongoing training and continuous education in the name of health care access, patient benefit, needed change, and the common good.

This volunteer army of advocates can train and prepare for research advocacy through numerous mechanisms. Many are drawn to advocacy through personal or family experience with cancer. They must however be motivated to become fully participatory through several initiatives, including: (i) individual study; (ii) advocacy group interaction and training; (iii) local, national, and international programs focused on research training and networking; (iv) attendance and participation at scientific meetings; and (v) online resources if available. This list is not comprehensive, and individuals and groups can explore additional opportunities for research advocacy training. For example, the Scientist-Survivor Program held at the American Association for Cancer Research Annual meeting is designed to build partnerships between scientists and patients/survivors. It “exposes advocates to special lay-language lectures, small group discussions and other interactions that provide a solid background in cancer research” [7]. The program offers an excellent training opportunity, and advocates from all over the world participate and network through the program.

Once trained, research advocates will grow in proficiency and influence, providing patient perspectives and keeping the focus firmly on patients. They can serve on ethics committees, concept and protocol review panels, research teams, grant funding panels, and other research-focused groups. While their opportunities and responsibilities may vary across the continent, within countries, regions, and ethnic groups, research advocates are important in the research process. Thus, they should be willing to share their stories (primarily focusing on the collective experience of patients and survivors), be persistent but not longwinded or self-important in providing the patient point-of-view, demonstrate deep concern and passion without conveying displaced anger and resentment, and build relationships rather than mark territory. They must stay focused on optimal patient benefit and outcomes.

In focusing on patient benefit and outcomes, research advocates concentrate on specific lines of inquiry and often frame their comments and concerns as questions. For example, they might ask:

• What can this research mean in terms of care, quality of life, and/or survival? Could the results change practice or add treatment options?

• Does the trial include provisions to overcome possible barriers to patient participation such as distance from the trial site, frequency of visits, incidental costs, and language and culture-related issues?

• How tolerable are the side effects of the experimental drugs and/or procedures?

• Outside of the clinic or hospital, will the drug be easy or practicable to take or administer?

• Will trial participants be able to work and/or take care of home responsibilities and remain on the trial?

• Who will supply the experimental drugs? Will there be a cost to trial participants?

• Who and what (groups, documents, and procedures) will protect the interests and safety of trial participants? Do you know who from the family or cultural group must be present to make the decision about trial participation?

No one is better positioned to ask such questions or has more at stake than patients and their advocates. Of course, the actual questions asked and areas of focus will be specific to local and population-specific concerns and realities.

### The feasibility of research advocacy in Africa

The formation and first-year efforts of the African Cancer Advocates Consortium (ACAC) answer the question of whether a viable African advocacy network is possible. It was formed, with 51 charter members, after the “International Workshop on Cancer Advocacy for African Countries” conducted during the 2011 AORTIC Conference. In its first year, ACAC members have remained engaged working on several initiatives. For example, ACAC members were instrumental in providing case studies for the “*Cancer Advocacy Training Toolkit for Africa*” published by AORTIC, the African Oxford Cancer Foundation (AfrOx), the European Society for Medical Oncology, and the Union for International Cancer Control [8]. In addition, the ACAC leadership proactively alerts African advocates about advocacy education and training opportunities. ACAC is the beginning of a robust network, with regional and subspecialty representation (including research advocacy). In a message to “Toolkit” users, David Kerr, founder and trustee at AfrOx, noted, “One of the most important ways we feel we can help to reduce the burden of cancer in Africa is to work with African cancer advocacy organizations to help educate and advocate about cancer in their countries” [9].

Research advocacy is both feasible and doable. Active existing advocacy groups, AORTIC support and workshops that provide training across advocacy subspecialties, the regionally and subspecialty diverse ACAC model, and the “Toolkit” serve as early evidence.

### Urgent needs for forward movement

Among the most urgent needs in developing sustainable and robust research advocacy in Africa are:

1. Public awareness efforts that put cancer on the priority list of health issues in Africa.

2. A research structure that supports and embeds research advocacy in its structure as a core value-added component.

3. Active recruitment and development of a core and pipeline of interested patients and other activists.

4. Ongoing training programs built, in part, through collaboration and shared cornerstones among advocacy groups across the continent and from other regions of the world.

5. Global partnerships among advocates, researchers and clinicians.

### The power of collective action and networking: advocacy experience

**Figure 1:**
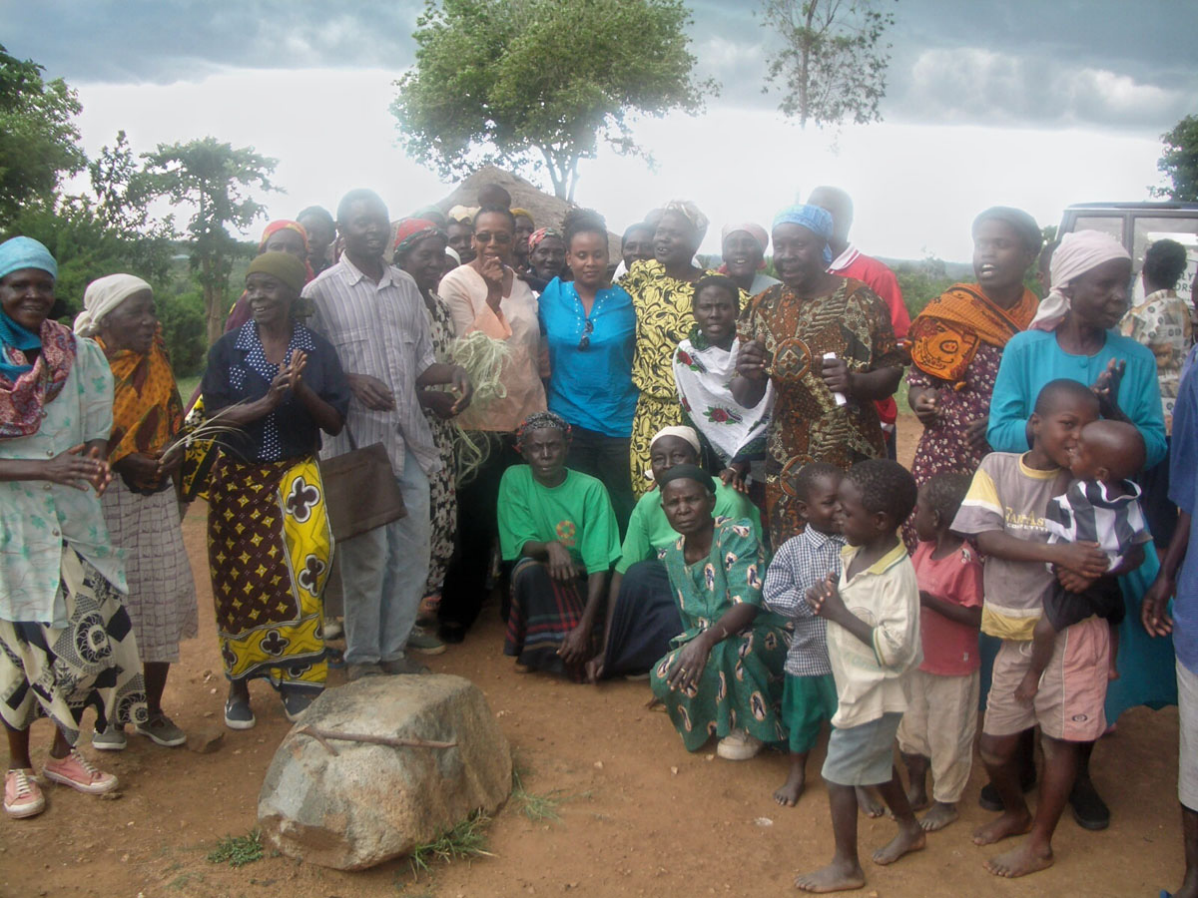
**The power of collective action** An HIV/AIDS Women’s Collective in a Kenyan village that supports its members through basket weaving and educates others through plays on HIV/AIDS. The spirit and dedication of the group demonstrates the power of collective action.

In 2005, while traveling in Kenya, the author and her daughter Nneka spent time in an HIV/AIDS Collective in a rural village (Figure 1). The vast majority of its residents are HIV-positive women and young children, with most of the husbands, fathers, and other adult males having died from AIDS or AIDS-related complications. To support the village, the surviving women sell lovely baskets they weave. In an environment perhaps hostile and unwelcoming, these courageous women also develop and present plays to educate others about HIV/AIDS and their experiences living with the infection or the disease. This lovely ensemble gave a special performance for the four purposeful travellers who made up our party, demonstrated the use of PUR (a tablet used to purify water from a pond they share with animals as a common drinking source), cooked a simple but filling meal (without request, providing the four of us with forks as all others ate communally by hand), and honored us with their generosity and hospitality. We asked for nothing, and they gave their all to us and the community.

The connection of this experience to the creation, growth, and influence of patient advocates and research advocacy is clear to the author. Without fanfare, this village demonstrates the power of collective action and networking. And, it is an illustration of the author’s belief that “Nothing is beyond the reach of a group of dedicated, passionate people…anywhere or under any circumstance on the planet.” Patient advocates—including research advocates—can be one such dedicated, passionate force, changing the landscape of cancer in Africa and working to defy the dire cancer incidence and death projections in this region of the developing world.

## Conclusions

The case for research advocacy in Africa is clear, and the framework suggested is usable although not necessarily the shape that research advocacy will take in countries and regions of Africa. In short, the framework includes the creation of a partnership between advocates and researchers, public and private support and investment, a research system that values patient input and embeds research advocacy, a pipeline of individual citizens willing and able to be (become) research advocates, judicious borrowing from and tailored use of already established models and concepts, and ongoing training and preparation. But the devil, of course, will be in the details and in the doing.

Advocates, through whatever subspecialty they engage, can make a difference in cancer awareness and prevention, incidence, care, and outcomes across Africa. In fact, they have already begun to do so. The only qualifier is the requirement for action, that is, moving beyond the rhetoric of urgency, recommendations, grand statements to practical steps, assignments, reasoned collaborations, pilot projects, and implementation. These are all feasible, all doable, all necessary.

## List of abbreviations used

AORTIC: African Organisation for Research and Training in Cancer; ACAC: African Cancer Advocates Consortium; AfrOx: African Oxford Cancer Foundation; CBPR: Community-Based Participatory Research; NCI: National Cancer Institute.

## Competing interests

The authors declare that she has no competing interests.

## Author’s contribution

MJS wrote the article.

## Supplementary Material

Additional file 1 **A tribute to patients and survivors** This is a tribute to cancer patients and survivors who give so much of themselves even as they struggle through their own cancer journeys. In 2005, the author and her daughter Nneka Scroggins planted a tree on the Masaai Mara in Kenya in honor of cancer survivors everywhere. Click here for file

## Declarations

The authors declare that funding for publication of the article was funded by an award provided by the National Cancer Institute (Award Number R13CA162899).

This article has been published as part of *Infectious Agents and Cancer* Volume 8 Supplement 1, 2013: Selected articles from The International Workshop on Cancer Advocacy for African Countries (CAAC) 2011. The information provided in this paper was also presented at the “Cancer in Africa: Building Transnational Research Collaborations” conference in London, England, in September 2012. The full contents of the supplement are available online at http://www.infectagentscancer.com/supplements/8/S1 The Supplement Editors declare they have no competing interests.
