# Supplementary figures and images for: Perspective of an advocate: a case and framework for research advocacy in Africa
Source: Infect Agent Cancer. 2013 Jul 15;8(Suppl 1):S4. doi: 10.1186/1750-9378-8-S1-S4 (PMC3716697; doi:10.1186/1750-9378-8-S1-S4)

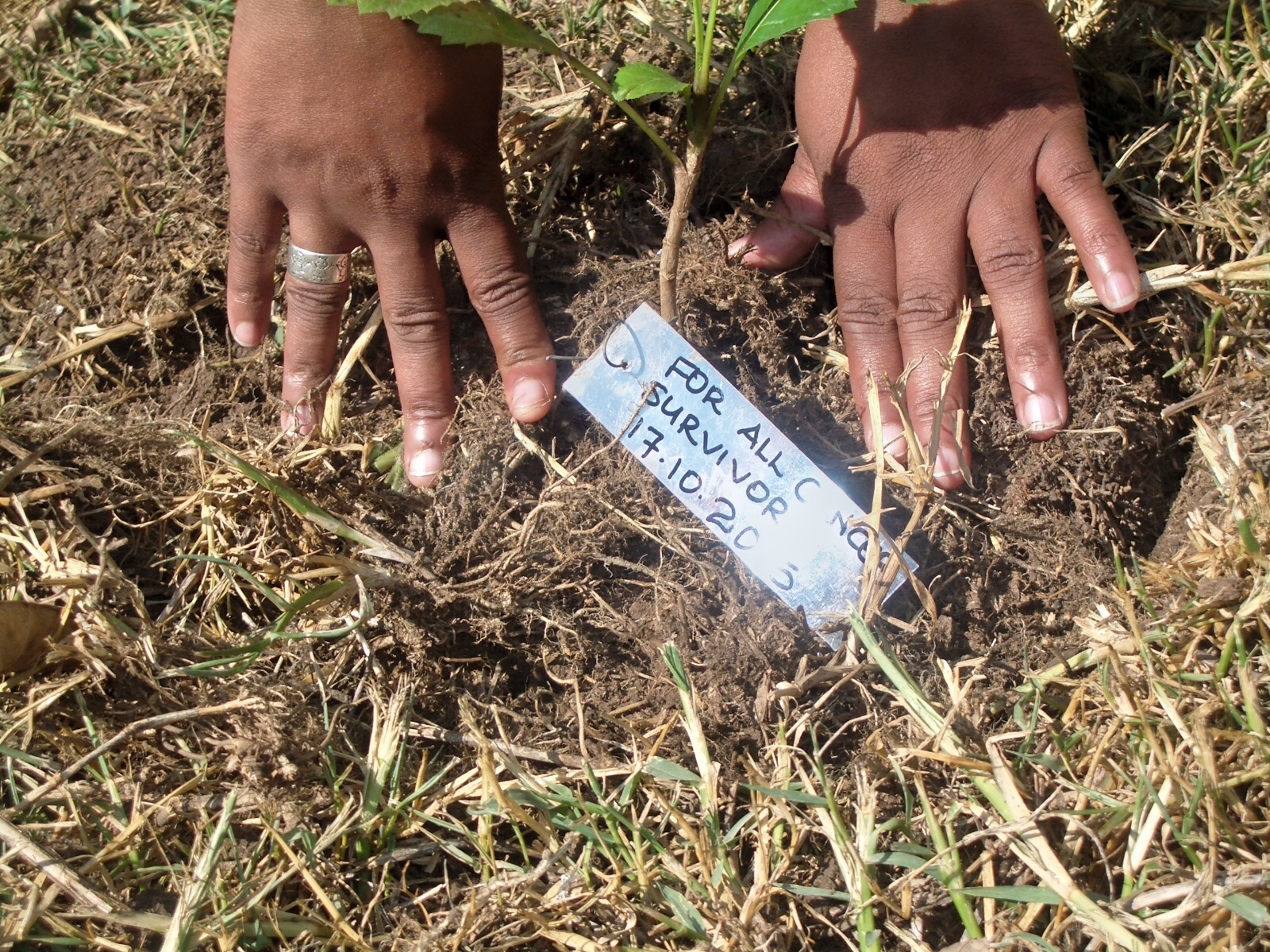

Supplement: Additional file 1 — A tribute to patients and survivors This is a tribute to cancer patients and survivors who give so much of themselves even as they struggle through their own cancer journeys. In 2005, the author and her daughter Nneka Scroggins planted a tree on the Masaai Mara in Kenya in honor of cancer survivors everywhere. [file 1750-9378-8-S1-S4-S1.JPG]
